# Coprophagous features in carnivorous Nepenthes plants: a task for ureases

**DOI:** 10.1038/s41598-017-11999-z

**Published:** 2017-09-14

**Authors:** Ayufu Yilamujiang, Anting Zhu, Rodrigo Ligabue-Braun, Stefan Bartram, Claus-Peter Witte, Rainer Hedrich, Mitsuyasu Hasabe, Caroline R. Schöner, Michael G. Schöner, Gerald Kerth, Célia R. Carlini, Axel Mithöfer

**Affiliations:** 1 Department of Bioorganic Chemistry, Max Plank Institute for Chemical Ecology, 07745 Jena, Germany; 2 Institute of Plant Nutrition, Leibniz University Hannover, 30419 Hannover, Germany; 3 Center of Biotechnology, Universidade Federal do Rio Grande do Sul, 91501-970 Porto Alegre, Brazil; 4 Institute for Molecular Plant Physiology and Biophysics, University of Würzburg, 97082 Würzburg, Germany; 5 National Institute for Basic Biology, Okazaki, 444-8585 Japan; 6 Zoological Institute and Museum, Ernst-Moritz-Arndt-Universität Greifswald, 17489 Greifswald, Germany; 7 Brain Institute (BRAINS-InsCer), Pontifícia Universidade Católica do Rio Grande do Sul, 90610-000 Porto Alegre, Brazil

## Abstract

Most terrestrial carnivorous plants are specialized on insect prey digestion to obtain additional nutrients. Few species of the genus *Nepenthes* developed mutualistic relationships with mammals for nitrogen supplementation. Whether dietary changes require certain enzymatic composition to utilize new sources of nutrients has rarely been tested. Here, we investigated the role of urease for *Nepenthes hemsleyana* that gains nitrogen from the bat *Kerivoula hardwickii* while it roosts inside the pitchers. We hypothesized that *N. hemsleyana* is able to use urea from the bats’ excrements. In fact, we demonstrate that ^15^N-enriched urea provided to *Nepenthes* pitchers is metabolized and its nitrogen is distributed within the plant. As ureases are necessary to degrade urea, these hydrolytic enzymes should be involved. We proved the presence and enzymatic activity of a urease for *Nepenthes* plant tissues. The corresponding urease cDNA from *N. hemsleyana* was isolated and functionally expressed. A comprehensive phylogenetic analysis for eukaryotic ureases, including *Nepenthes* and five other carnivorous plants’ taxa, identified them as canonical ureases and reflects the plant phylogeny. Hence, this study reveals ureases as an emblematic example for an efficient, low-cost but high adaptive plasticity in plants while developing a further specialized lifestyle from carnivory to coprophagy.

## Introduction

A striking feature of plants is the ability to adapt with high flexibility to completely different ecological environments and to survive even in extreme habitats. Some plants that live in nutrient poor environments evolved carnivory to obtain nitrogen, phosphorus and minerals from animals. Various trapping mechanisms exist in carnivorous plants such as pitfall traps (*Nepenthes* spp.), adhesive traps (*Drosera* spp.), snap traps (*Dionaea muscipula*), corkscrew traps (*Genlisea* spp.), and suction traps (*Utricularia* spp.) supporting a broad spectrum of prey selection^1^. In all cases, the different traps derived from metamorphosis of leaves forming new adaptive organs^1^. Carnivorous plants attracted attention of scientists for centuries, including Charles Darwin, who already in 1875 reported pioneer suggestions on plant carnivory^2^. In recent years, proteomic and molecular approaches provided many new insights into plant carnivory^3–8^. The more we learn about carnivory in plants the more we realize that its basis relies on the plants’ ability to defend themselves against attackers such as herbivorous insects or microbes. In a simplified view we may state that carnivorous plants use already existing pathways and strategies, from signaling to hydrolytic, defense-related enzymes, and transferred these mechanisms into a different ecological context, i.e. carnivory (e.g. in *Dionaea* ^3^; *Drosera* ^6, 7^; *Nepenthes* ^5, 8^, *Cephalotus* ^4^).

A taxon of carnivorous plants Darwin never worked with is the genus *Nepenthes*. This genus contains more than 120 species; all of which possess pitcher-shaped leaves that are filled with a digestive fluid. In most of the *Nepenthes* species, these pitchers serve to attract, capture and digest arthropod prey, and take up animal-derived nutrients. However, several of the *Nepenthes* species further developed alternative strategies for nutrient acquisition often based on mutualistic interactions with animals. *Nepenthes bicalcarata*, for example, hosts the ant *Camponotus schmitzi*, which contribute more than 50% to the plant’s foliar nitrogen with waste material, mainly feces^9^. Such interactions can also be found between *Nepenthes* species and mammals. The montane species *Nepenthes lowii, Nepenthes rajah* and *Nepenthes macrophylla* benefit from fecal nitrogen of small mammals such as the mountain shrew, *Tupaia montana* that are rewarded with nectar from the pitchers’ lid^10^. Similarly, individuals of the bat species *Kerivoula hardwickii* defecate into pitchers of *Nepenthes hemsleyana* while roosting inside^11, 12^. Thus, the visiting activity of mutualistic mammalian partners significantly increase foliar nitrogen content in the host plants, *N. hemsleyana* and *N. lowii* ^10–12^. However, the precise origin of the nitrogen and the biochemical background these plants use to harvest the external nitrogen trapped inside the pitchers remained unclear, so far.

Here we demonstrate that *Nepenthes* pitchers are able to absorb and use the main component of bat excrement, urea. This finding disproves the statement of Darwin who wrote that urea cannot be used by carnivorous plants, at least in *Drosera* ^2^. However, in contrast to arthropods, which excrete uric acid, mammals are ureotelic and excrete urea. Because urea is an extremely stable molecule, a urease is necessary and involved as a key enzyme in urea degradation thereby releasing nitrogen in the form of ammonia. From two *Nepenthes* species the respective urease gene was cloned and the one from *N. hemsleyana* was functionally expressed. Ureases from *Nepenthes* and five other carnivorous plants from different genera are phylogenetically compared to ureases from non-carnivorous plants and fungi. The results suggest that for carnivorous plants it was not necessary to develop a specific urease for establishing a coprophagous lifestyle in *Nepenthes* but to recruit a reliable and available enzyme into an adapted nutritional context.

## Results

### Urea Uptake and Metabolism in Nepenthes

**Figure 1:**
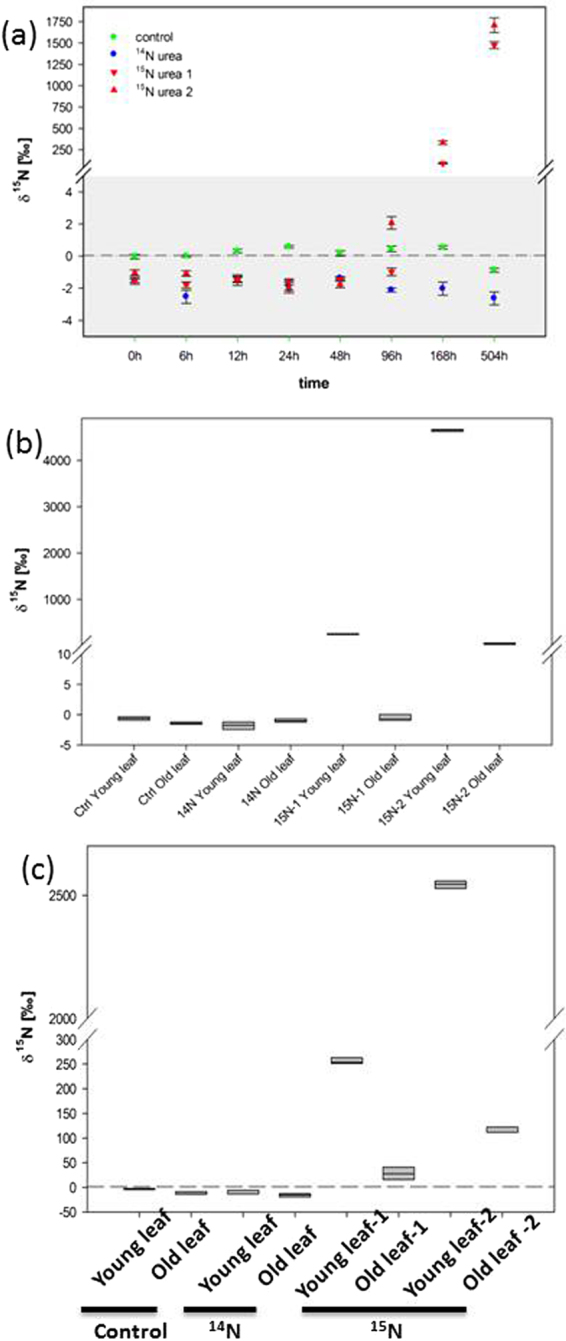
Nitrogen uptake, mobilization and incorporation in *Nepenthes*. (**a**) ^15^N uptake in leaf-base of ^15^N-enriched urea-fed pitcher. ^15^N-enriched urea was injected into the closed *Nepenthes alata* pitcher and ^15^N content in the leaf base of injected pitchers was analyzed after 0, 6, 12, 24, 48, 96, 168 and 504 hours by isotope-ratio mass spectroscopy (IRMS). (**b**) Nitrogen mobilization from pitcher to the different leafs in the same branch. ^15^N-enriched urea was injected into the closed *N. alata* pitcher followed by the analysis of ^15^N content in younger and in older leaves of the same branch after 3 weeks by IRMS. Leaves 1 and 2 represent individual leaves in independent experiments. (**c**) ^15^N incorporation into protein in *Nepenthes*. ^15^N-enriched urea was injected into the closed *N. alata* pitcher. ^15^N content in the protein extracts of young and old leaves of the same branch was analyzed after 3 weeks by IRMS. ^14^N-fed and not fed plants were used as a control.

In the mutualism between bats and *Nepenthes*, the pitcher plant is provided with high amounts of urea^11, 12^. The urea-derived nitrogen is suggested to enter nitrogen metabolism in *Nepenthes* to drive growth and development^12^. To test whether or not *Nepenthes* species are generally able to take up and metabolize urea, ^15^N-enriched urea was fed to pitchers of *N. alata*, a carnivorous relative of *N. hemsleyana* that derives nitrogen from captured insects. The distribution of ^15^N was analyzed by isotope ratio mass spectrometry (IRMS). Upon ^15^N-enriched urea application directly into the fluid of still closed pitchers, a slow time dependent increase of ^ 15^N was measured in the leaf-base of the treated pitcher starting after 96 h (Fig. 1a). An increase of ^15^N was detectable for old as well as for young leaves sitting at the same branch of the plant (Fig. 1b). The finding that developing leaves received more ^15^N nitrogen compared to older leaves confirms earlier suggestions that nitrogen is preferentially directed towards developing sinks^13^ (Fig. 1b). Our data document that *Nepenthes* is able to take up urea from the pitcher and to allocate this feces-derived nitrogen to non-pitcher tissues^10, 11^. In plants, nitrogen released from urea hydrolysis into ammonia and CO_2_ is subsequently incorporated into macromolecules. In line with this assumption, protein extracts from leaves in the same branch as the^15^N-enriched urea-fed pitcher exhibited higher ^15^N/^14^N ratios (Fig. 1c).

### Cloning, Phylogeny and Expression of Urease from *Nepenthes*

**Figure 2:**
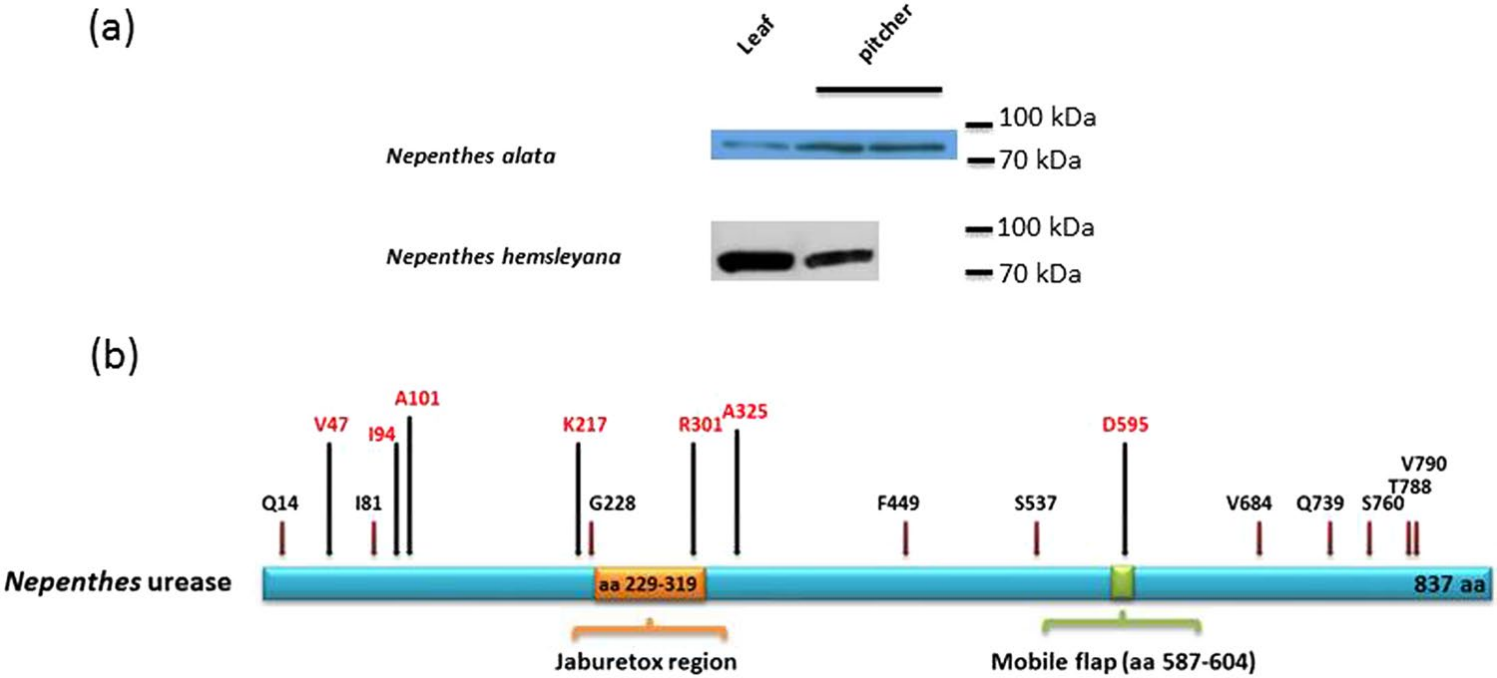
Features of urease from *Nepenthes*. (**a**) Assessment of urease protein in crude protein extracts from *N. hemsleyana* and *N. alata* by immunoblot using polyclonal anti-jackbean urease antibodies (for comparison and the full-length gels see Fig. S3). (**b**) Schematic illustration of *Nepenthes* urease based on its amino acid (aa) sequence analysis. Active-site mobile flap and jaburetox region of *Nepenthes* urease are indicated in different colors. Positions of amino acids found to be unique for carnivorous plants’ ureases when compared with *Canavalia ensiformis, Glycine max*, and *Arabidopsis thaliana* are indicated by arrows with red label; amino acids found to be unique only for *Nepenthes* ureases are indicated by arrows with black label. In this case amino acids are indicated only when all non-*Nepenthes* species show an identical amino acid in that position.

Using a polyclonal jackbean urease antiserum for immunoblotting, in crude protein extracts from leaf and pitcher tissues of both *N. hemsleyana* and *N. alata* a cross-reacting protein was detected (Fig. 2a). Enzymatic urease activity, however, was not detectable in the pitcher fluid but measurable only in leaves extracts from *N. hemsleyana* (35 nmol NH_3_ min^−1^ g fw^−1^) and *N. alata* (20 nmol NH_3_ min^−1^ g fw^−1^).

Based on the sequences of plant urease genes, degenerate primers were designed and used for a PCR approach in *N. alata* and *N. hemsleyana*. The two full-length coding sequences for the urease genes from both *Nepenthes* species were cloned (each 2,514 bp long). They encode a single polypeptide chain with the basic characteristics of known plant ureases such as an active site that includes a nickel binding site and a jaburetox domain^14^ (Fig. 2b). Both deduced protein sequences consist of 837 amino acids (predicted molecular mass of the proteins: *N. hemsleyana* 90.0 kDa and *N. alata* 89.6 kDa). Sequence comparison of *Nepenthes* ureases with those from other sources indicates highly conserved amino acid residues in their catalytic sites (Fig. S1). When compared with the well-studied and crystallized *Canavalia ensiformis* urease (jackbean urease, JBU)^15^, the catalytic-site residues in *Nepenthes* ureases are as follows (*Nepenthes/Canavalia*): H404/407, H406/409, K487/490, H489/492, D491/494, H516/519, H542/545, C589/592, H590/593, R606/609, D630/633, and A633/636. The active sites consist of a bi-nickel center coordinating two nickel ions. Typically, K487/490 can be carbamylated and acts as a bridging residue between the two nickel ions^15^. We found this Lysine in all sequences of carnivorous and non-carnivorous plants investigated here (Fig. S1); therefore the catalytic properties of all ureases are likely similar. The amino acid residues that are involved in the architecture of the active site build part of a mobile flap, which acts as a gate for the substrate. In JBU, this mobile flap ranges from amino acids M590 to H607^15^. In *Nepenthes* ureases the corresponding region is also present and spans from M587 to E604 including an essential C589 (C592 in JBU) residue (Fig. 2b). Precisely in this region one amino acid, in *Nepenthes* D595, was found only in all the seven carnivorous plants included in this study, but was changed in the two legume species studied and in *Arabidopsis thaliana*. In total, the urease sequences show seven of such convergent carnivorous plant-specific amino acid substitutions (Figs 2b; S1). Ten further amino acid changes were found to be unique for the two *Nepenthes* species (Figs 2b; S1).

The whole amino acid sequences of the carnivorous *N. alata* and the coprophagus *N. hemsleyana* ureases show more than 97% sequence identity at the amino acid level (Table S1). In addition, the amino acid sequences of the two *Nepenthes* ureases share ≥86% similarity and ≥75% identity to ureases from other carnivorous plants and even to non-carnivorous plants such as *Glycine max* and *Canavalia ensiformis* (Table S1). Within carnivorous plants only the urease of the endemic species *Genlisea aurea* is slightly different and shows less identity to the other ureases analyzed (Table S1). With the exception of *Cephalotus follicularis*, the level of urease sequence identity of all the carnivorous plants is slightly lower when compared with the three non-carnivorous species.

**Figure 3:**
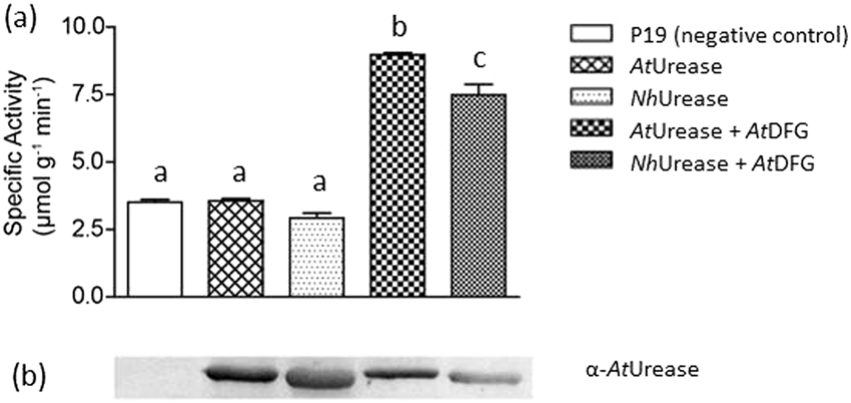
Expression and enzymatic activity of heterologous ureases in *Nicotiana benthamiana*. Proteins were transiently expressed in *Nicotiana benthamiana* for six days, afterwards total proteins of the plants were extracted and desalted. (**a**) Specific urease activity of: non-transformed *Nicotiana benthamiana*, P19, negative control; *A. thaliana* urease alone, *N. hemsleyana* urease alone, *A. thaliana* urease with accessory proteins UreD, UreF and UreG, *N. hemsleyana* urease with accessory proteins UreD, UreF and UreG. Error bars are SE (n = 3) (Different letters label groups which are significantly different (p < 0.05; Oneway Anova with Turkey’s post-hoc test). (**b**) Assessment of urease proteins in the corresponding samples by immunoblot employing anti- *A. thaliana* urease-specific antiserum (for comparison and the full-length blot see Fig. S4).

In order to demonstrate the functionality of the cloned urease of *N*. *hemsleyana*, designated as *NhUrease*, it was transiently expressed in *Nicotiana benthamiana*, a system that has a weak urease background and that was successfully used before^16, 17^. After six days, urease activity was measured in total plant crude extracts. To gain function, plant ureases depend on accessory proteins that help the insertion of nickel ions into their active site^16–18^. As expected, in absence of the accessory proteins the *NhUrease* had no enzymatic activity. Only upon co-expression of three urease accessary proteins from *A. thaliana*, UreD, UreF, and UreG, *NhUrease* gained function (Fig. 3). The successful expression of the accessory proteins was shown exemplarily for UreD using a *At*UreD antiserum^17^ (Fig. S2). *A. thaliana* urease expressed in the same *Nicotiana benthamiana* system as “positive control” showed comparable results (Fig. 3).

**Figure 4:**
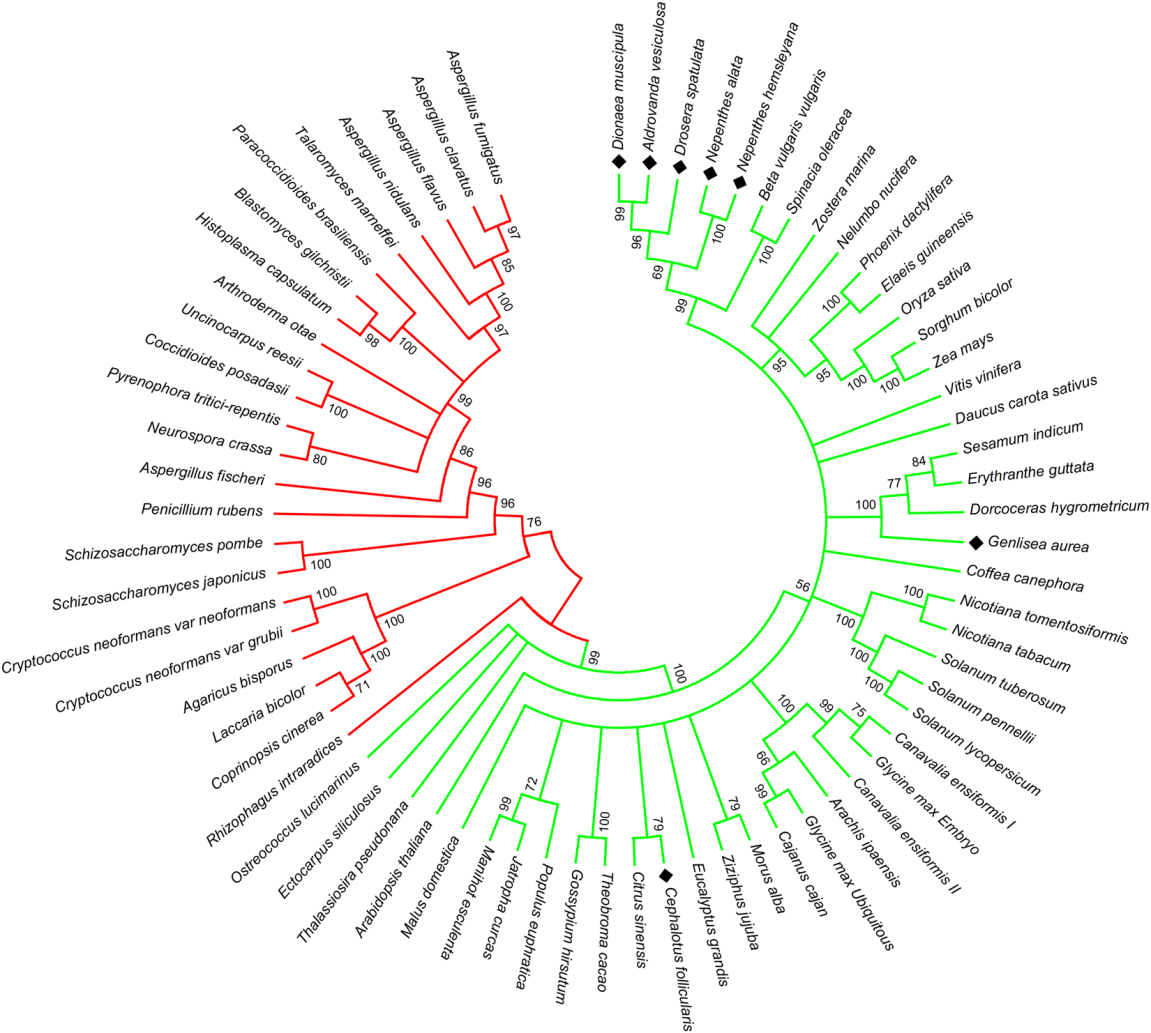
Phylogenetic analysis of ureases. Molecular phylogenetic analysis of plant (green) and fungal (red) ureases, with diamonds highlighting ureases from carnivorous plants included in this study. The tree with the highest log likelihood is shown. Branchings under 50% of bootstrap support (1,000 pseudoreplicates) were collapsed.

To learn more about a putative functional evolution of ureases in *Nepenthes* and other carnivorous plants’ taxa, a comprehensive phylogenetic analysis for eukaryotic (fungal and plant) ureases was conducted to construct an evolutionary tree (Fig. 4). The ureases from *N. alata* and *N. hemsleyana, Dionaea muscipula*, *Drosera spatulata*, and *Aldrovanda vesiculosa* were found closely related (Supplementary Table 1; Fig. 4) forming part of a separate clade. This clade, however, does not contain ureases from carnivorous *Genlisea aurea* ^19^ or *Cephalotus follicularis*, which are found in separated clades (Fig. 4). For the carnivorous *Utricularia gibba* no urease gene was detectable (RefSeq NC_021449.1)^20^.

## Discussion

Our results suggest that carnivorous plants did not need to develop a specific urease for establishing a coprophagous lifestyle in *Nepenthes* but to recruit a reliable and available enzyme into an adapted nutritional context. On the cellular level, the main function of plant ureases is related to nitrogen recycling from endogenous urea. The presence of ureases in almost all plant taxa indicates the vital role of this enzyme in metabolism, as part of arginine catabolism from which arginase generates ornithine and urea^21^. In most terrestrial vertebrate animals urea is a metabolic waste product and is removed from the body in form of urine^22^. More than 70% of bats’ urine dry weight consists of urea^23^. We could show that carnivorous *Nepenthes* can harvest nitrogen in the form of urea “captured” in their pitchers and released upon cleavage by the plant’s urease. Thus, by employing urease not only for nitrogen recycling, *Nepenthes* plants acquired a new source for nutrient supplementation. Our studies with ^15^N-enriched urea injected into closed pitchers demonstrated that the exogenous urea is taken up from the pitcher fluid, hydrolyzed and the nitrogen is distributed within the plant. The pitcher fluids of *N. alata*, and several other species of *Nepenthes* grown under the described conditions, were shown to be sterile and unsuitable for microbial growth^24^. This and that fact that we could only detect a urease and its activity in pitcher tissue but not in the fluid makes a possible microbial involvement in urea hydrolysis unlikely. The absence of urease also prevents any alkalinization of the pitcher fluid by released ammonia from hydrolyzed urea, which would work against the plants’ effort keeping the pitcher fluid acidic. Also, in contrast to other *Nepenthes* enzymes found in the pitcher fluid^25–27^, no signal peptides were detectable for the urease protein sequences, which makes secretion into the digestive fluid unlikely. Thus, considering that the presence of urease in the pitcher fluid was excluded, the involvement of a urea transporter is probable. Urea transporters are present in most organisms and belong to different classes, some functioning as passive channels, others as secondary active transporters. Interestingly, urea transporters commonly found in animals and bacteria (UT, UreI, Yut) are absent in plant and fungal genomes^28^. These latter genomes encode a different family of high affinity urea transporters, DUR3^29^. In addition, passive transport of urea can be mediated by aquaporins. These channels can conduct certain low molecular weight solutes, including urea, along their concentration gradient through the membrane^21^. Whether or not *Nepenthes* pitcher tissue has DUR-type transporters and/or aquaporins for urea transport and uptake remains to be addressed in further studies.

Based on our results that a functional urease is present in the carnivorous *N. alata*, we claim that urease supported a further transition from carnivory to coprophagy in the closely related *N. hemsleyana* and probably in other coprophagous *Nepenthes* such as *N. lowii*. In carnivorous plants, functional diversification of genes and proteins, which act in quite different pathways, has been described. For example, class I chitinases are pathogenesis-related hydrolytic enzymes in higher plants that are involved in defense against herbivory and pathogen attack^30^. Interestingly, based on sequence homology, for carnivorous plants belonging to the order Caryophyllales a functionalization of class I chitinases has been demonstrated supporting the idea of carnivorous specialization. In that case, two chitinase I subclasses evolved, very likely due to different substrate degradation requirements in the chitin of fungi and insects. Thus, subclass Ia is still related to pathogen defense and sub-class Ib is related to carnivory^31^. Recently, the same chitinase subclass Ib has also been found in *Drosera* ^32^. Even more striking are certain ribonucleases in carnivorous plants. While S-like RNases are usually induced by stress such as low nutrition level, feeding damage or even senescence, in carnivorous plants S-like ribonucleases evolved to function in carnivory^33^. Surprisingly, with respect to the conventional phylogeny, the S-like ribonucleases from phylogenetically non-related carnivorous plants such as *C. follicularis* on one side and *Nepenthes, Dionaea* and *Drosera* on the other side, show higher similarities than expected. This phenomenon was explained based on a carnivory-dependent evolution of the enzyme^34^. Concerning the urease in *Nepenthes*, we did not find evidence of such specialization at the sequence level although convergent amino acid changes for the carnivorous plants could be detected (Fig. 2b), comparable with results found for S-like ribonucleases^34^, chitinases and phosphatases^4^.

Regarding the evolutionary age of ureases in plants, they are generally considered to be as old as plants; themselves nevertheless, a putative horizontal transfer of prokaryotic origin to unicellular algae is suggested^35^. The phylogenetic tree constructed in our work, including seven species of carnivorous plants, is in agreement with the tree encompassing ureases from all domains of life^35^. It is also in agreement with the plant phylogeny, as confirmed by comparison with the Interactive Tree of Life using the PhyloT tool from iTOL 3.0^36^. Thus, the distant positions of *G. aurea* and *C. follicularis* ureases found in the tree – compared with the “*Nepenthes* clade” – were not surprising (Fig. 4). Interestingly, no urease was found in the genome of the carnivorous plant *Utricularia gibba*. Indeed, it has been shown that plants from this genus rely on their traps to acquire phosphates and possibly sulfates^20, 37^. Considering that nitrogen is not a limiting nutrient for this group of carnivorous plants, and that urease is not essential for nitrogen metabolism, the absence of this enzyme in such a heavily reduced genome as found for *U. gibba* ^20^ is comprehensible and somewhat expected.

Our findings suggest that in carnivorous plants ureases, in contrast to other enzymes such as chitinases^31^, did not further evolve towards a specialized function but carry out their inherent enzymatic activity. This was not surprising in view of the fact that the substrate urea is clearly defined, in contrast to e.g. chitins from insect and fungi. In addition, carnivorous plants only rarely come in contact with urea. Typically, insects are used as prey. Even if these uricotelic animals defecate into/onto a trap no urea but uric acid is provided. That also applies to those few specialized cases where carnivorous or proto-carnivorous plants, respectively, use feces from interacting arthropods to obtain nitrogen^9, 38–41^.

In the pitchers of *N. hemsleyana* plants only the employment of urease allows to metabolize the bat-derived, exogenously available urea. In general, the switch from carnivory to coprophagy seems to be connected to several adaptations on the plant side to attract and host their mammal partners, such as the shape of the pitchers or color/acoustic patterns that indicate the plants’ presence to the mammals^42–45^. In contrast, this study suggests that for parts of the digestive processes no additional adaptations have been necessary, which indicates reduced costs for the switch in nutrient acquisition strategies. As a consequence, based on basic biochemical features, a specialized coprophagous lifestyle could be established in at least few *Nepenthes* species. This progression can be seen as a kind of niche segregation within the botanical-carnivory-niche in order to avoid competition with other *Nepenthes* species from the same habitat. Almost all *Nepenthes* species consume the same range of prey, with ants (up to 70%) representing the highest proportion of caught prey^46^. However, more studies are needed to fully understand the physiology of exogenously provided urea, first of all its uptake from the pitcher fluid, the nitrogen distribution, and its utilization within the pitcher plant. Nevertheless, at this point it is tempting to speculate that *N. hemsleyana* might be on the way to lose the ability of carnivory in favor of coprophagy, thereby becoming an example for progressive reduction.

## Methods

### Plant material and treatment

*Nepenthes alata* Blanco plants were grown in growth chambers at 20–25 °C, 80–85% humidity and a 16/8 h light/dark photoperiod. *Nepenthes hemsleyana* Macfarl. was grown in the greenhouse on a mixed substrate (sphagnum/bark/leaves/moss) with an average temperature of 23–25 °C and 80–100% humidity. The photoperiod was at least 12 h of light per day. For urea feeding experiments, 75 µl of 2 M urea (representing ~50 mM final concentration) was injected with a sterile syringe into one closed pitcher of *N. alata*. Either non-modified (^14^N) urea (Merck) or 10% ^15^N-enriched urea (Sigma-Aldrich) was used. ^15^N and ^14^N content in leaf tissue were analyzed after different time points by isotope-ratio mass spectrometry (see below). ^14^N urea-fed and not fed plants were used as controls.

### RNA isolation and cDNA synthesis

High quality total RNA was isolated from young *Nepenthes* pitcher tissue using Invitrap spin plant RNA Mini Kit (STRATEC) according to the manufacturer’s instruction. First-strand cDNA synthesis was performed using Superscript III reverse Transcriptase (Invitrogen) by using Oligo (dT)12-18 following the manufacturer’s protocol.

### Cloning of Nepenthes urease

Eight different plant urease genes (BAB78715.1, CAC43859.1, AAO85884.1, AAA83831.1, CAC43845.1, CAC43860.1, AAN08919.1, NP_176922.1) were aligned with MEGA (v5.5) using MUSCLE algorithm^47^. Based on the alignment degenerate primers (forward: AARAATGTNHTNCCBTCWTCAAC and revers: AGGWGTDGGDATRCTNSCATTT) were designed. PCR was performed using the designed degenerate primer. A fragment of around 500 nt was amplified using cDNA from *Nepenthes* as a template. Based on that sequence we designed specific primers to obtain the full-length sequence of *Nepenthes* urease by performing RACE-PCR. *5*′*-RACE*: First round of 5′-RACE was performed using gene specific primer1 (GSP1: TCAGAGTCAAGTGGCCCTCTCTGCACTT) and nested gene specific primer 1 (NGSP1: GCGACCCATAGCCTGTGAATCAGAAGAGA). A second round of 5′-RACE was needed since 5′-end was incomplete after first round 5′-RACE. Based on the sequence of the first round 5′-RACE product, gene specific primer 2 (GSP2: AGACAGGCAGCTGGCGGGTACCCAGA) was designed and 5′-end of *Nepenthes* urease was amplified. *3*′-*RACE*: 3′-RACE gene specific primer (3′GSP: TACGAGCCGAAACCATTGCTGCAGAAGACA) was designed based on the sequence of the first round 5′-RACE product. SMART RACE cDNA amplification kit (Clontech) was used. Template RNA for RACE-PCR was isolated as described above. All PCR products were cloned into pJET1.2/blunt plasmid and sequenced. A cDNA contig was formed with seqMan and open reading frame (ORF) was determined. The complete ORF of both *N. hemsleyana* and *N. alata* ureases were amplified and cloned into pJET1.2/blunt for sequencing. Proofed sequences were submitted to EMBL.

### Protein extraction, western blotting, heterologous expression, and urease activity detection

Proteins were extracted from *Nepenthes* leaf or pitcher tissue using 50 mM phosphate buffer (pH 7.5) containing 2% PVPP, 50 mM NaCl, 1 mM EDTA and 20 mM DTT (DTT was added fresh before extraction). Protein separation by SDS-PAGE and blotting were performed using Miniprotein TGX gels (Bio-Rad) and Trans blot turbo blotting system (Bio-Rad) respectively. Polyclonal anti-jackbean (Thermo Fisher Scientific), anti-*A. thaliana* urease, and anti-*A. thaliana* UreD antibodies^17^ were used for immunoblot urease detection and indicated in the particular experiments. Transient expression of *NhUrease* in *Nicotiana benthamiana* was performed according to^16, 17^. For functional tests, *NhUrease* and accessary proteins from *A. thaliana*, UreD, UreF, and UreG, were coexpressed. Urease activity was measured as described^48^.

### Isotope ratio mass spectrometry analysis (IRMS)

About 2 mg of dried and ground plant material was weighed with an ultra-micro balance (UMX2, Mettler-Toledo), in small 40 µl tin capsules (3.5 × 5 mm, HEKATech. HE 24005300). The capsules were sealed and combusted (oxidation at 1020 °C, reduction at 650 °C) in a constant helium stream (80 ml min^−1^) quantitatively to CO_2_, N_2_ and H_2_O using an elemental analyzer (EuroEA CN_2_ dual, HEKAtech). After passing a water trap (MgClO_4_) the gases were separated chromatographically at 85 °C and transferred via an open split to a coupled isotope ratio mass spectrometer (IsoPrime, Micromass). Isotope ratios were calculated as:

$${\delta }^{N}E=\frac{{R}_{sample}}{{R}_{standard}}-1$$

δ values usually are small numbers. Hence, they are commonly multiplied by 1000 and communicated in ‰ units or mUr^49^. R is the ratio of heavy to light isotope (^15^N/^14^N) of the sample and the standard, respectively. δ^15^N is the relative deviation of the heavy to light isotope ratio from the international standard (air-N_2_ for nitrogen). Samples were measured against our laboratory working standard alice-1 (acetanilide, δ^15^N = −1.44 ± 0.12‰) which has been calibrated for δ^15^N by a two-point normalization using IAEA reference material IAEA-N1 (+0.43‰) and IAEA-N2 (+20.40‰)^50^. Empty tin capsules were used as blanks. Three technical replicates of each plant material bulk sample were analyzed. A caffeine standard (δ^15^N = −4.01 ± 0.10‰) was analyzed together with the samples as QA reference material for long-term performance monitoring of the whole analytical setup; for details see^51^. δ^15^N values were not corrected for *m/z* = 30 (^15^N_2_) because in all samples the^15^N content was always below 2%. For isotope ratios of leaf proteins about 2 mg of acetone-precipitated protein were used.

### Phylogenetic analysis

Amino acid sequences of plant and fungal ureases were retrieved from the National Center for Biotechnology Information^52^ based on a previous urease phylogeny study by Ligabue-Braun and colleagues^35^. These sequences, along with the sequences of ureases from additional carnivorous plants presented here were aligned using MAFTT5^53^ and filtered for unreliable positions using Guidance2^54^. The final alignment was used to infer the evolutionary history of these ureases by using the Maximum Likelihood method, based on the LeGascuel2008 model. A discrete Gamma distribution was used to model evolutionary rate differences among sites, and the rate variation model allowed for some sites to be evolutionarily invariable. All positions containing gaps and missing data were eliminated. Significance was assessed via 1,000 bootstrap pseudoreplicates, and branchings under 50% of bootstrap support were collapsed. All evolutionary analyses were conducted in MEGA7^55^. Sequence similarity matrices were generated with MatGAT^56^.

## Data availability

The assembled amino acid sequences used for the phylogenetic analysis are available from: *Nepenthes alata*, Accession #: LT622248, EMBL; *Nepenthes hemsleyana*, Acc #: LT622249, EMBL; *Dionaea muscipula*, Acc #: comp223007_c0_seq. 3, http://tbro.carnivorom.com3; *Aldrovanda vesiculosa*, Acc #: KY293301, NCBI*; Genlisea aurea*, Acc #: EPS69592, NCBI; *Drosera spatulata*, Acc #: LC194217, NCBI; *Cephalotus follicularis*, Acc #: BDDD01005981 (gene region: 27762 to 34049)^4^. The data that support the findings of this study are also available from the corresponding author on request.

## Electronic supplementary material

Supplementary Information
