# Supplementary material for: Coprophagous features in carnivorous Nepenthes plants: a task for ureases
Source: Sci Rep. 2017 Sep 14;7:11647. doi: 10.1038/s41598-017-11999-z (PMC5599630; doi:10.1038/s41598-017-11999-z)
Supplement: Supplementary file 1 — Supplementary Information [file 41598_2017_11999_MOESM1_ESM.pdf]

Coprophagous features in carnivorous *Nepenthes* plants: a task for ureases

Ayufu Yilamujiang[1], Anting Zhu[2], Rodrigo Ligabue-Braun[3], Stefan Bartram[1], Claus-Peter Witte[2], Rainer Hedrich[4], Mitsuyasu Hasabe[5], Caroline R. Schöner[6], Michael G. Schöner[6], Gerald Kerth[6], Célia R. Carlini[3,7], Axel Mithöfer[1,]*

[1]Department of Bioorganic Chemistry, Max Plank Institute for Chemical Ecology, 07745 Jena, Germany.

[2]Institute of Plant Nutrition, Leibniz University Hannover, 30419 Hannover, Germany.

[3]Center of Biotechnology, Universidade Federal do Rio Grande do Sul, 91501-970 Porto Alegre, Brazil.

[4]Institute for Molecular Plant Physiology and Biophysics, University of Würzburg, 97082 Würzburg, Germany.

[5]National Institute for Basic Biology, Okazaki 444-8585, Japan.

[6]Zoological Institute and Museum, Ernst-Moritz-Arndt-Universität Greifswald, 17489 Greifswald, Germany.

[7]Brain Institute (BRAINS-InsCer), Pontifícia Universidade Católica do Rio Grande do Sul, 90610-000 Porto Alegre, Brazil.

Supplementary Information:

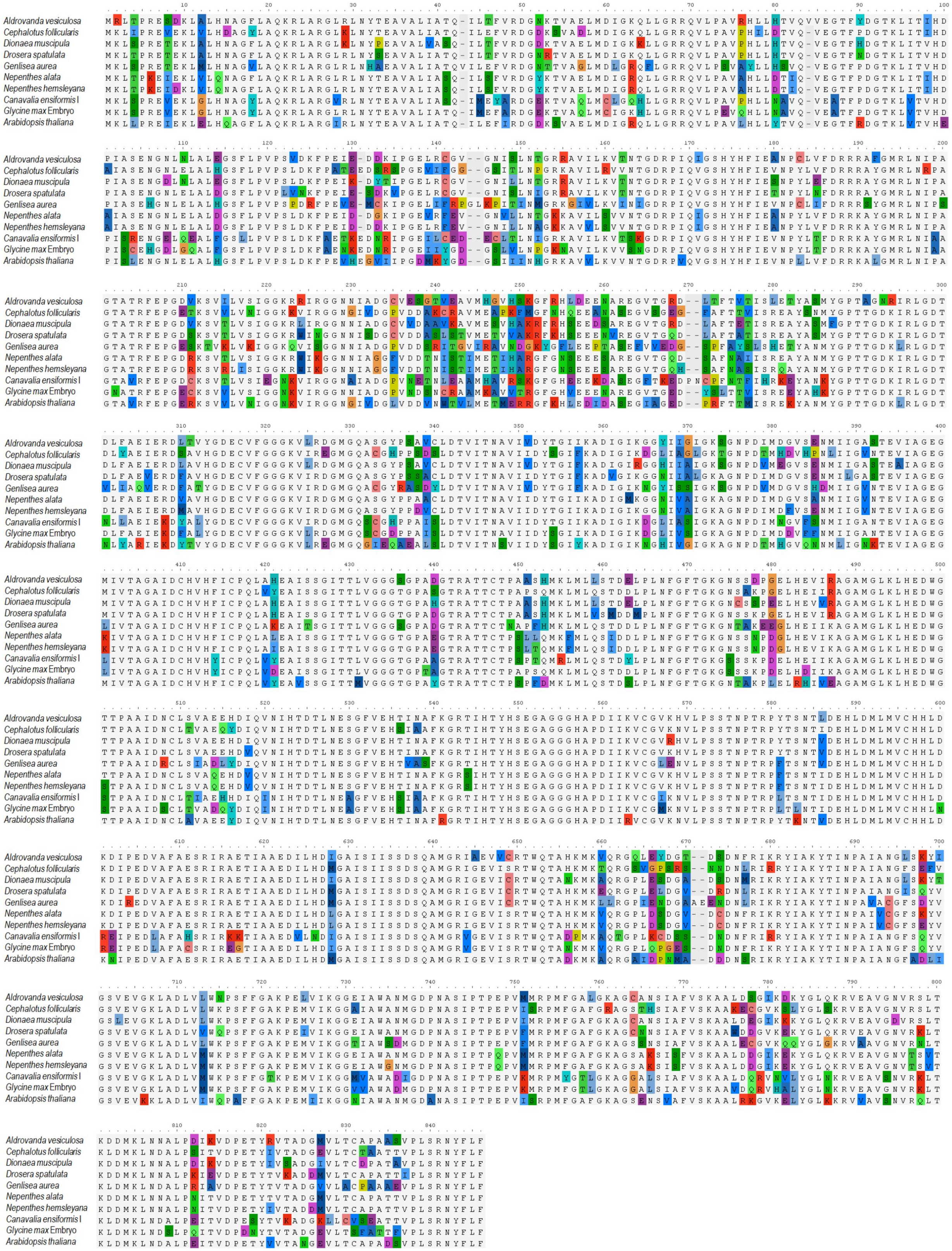
Aldrovanda vesiculosa
Cephalotus follicularis
Dionaea muscipula
Drosera spatulata
Genlisea aurea
Nepenthes alata
Nepenthes hemsleyana
Canavalia ensiformis I
Glycine max Embryo
Arabidopsis thaliana

Figure S1. Homology among amino acid sequences of ureases. Included are ureases from seven carnivorous plants (*Aldrovanda vesiculosa, Cephalotus follicularis, Dionaea muscipula, Drosera spatulata, Genlisea aurea, Nepenthes alata, Nepenthes hemsleyana*) and three non-carnivorous plants (*Canavalia ensiformis, Glycine max* embryo-specific*, Arabidopsis thaliana*). Different colors indicate individual amino acids that are different to the consensus amino acid at a particular position.

Table S1: Sequence similarities for model and carnivorous plant-derived ureases

	A. vesiculosa	*C. follicularis*	*D. muscipula*	*D. spatulata*	*G. aurea*	*N. alata*	*N. hemsleyana*	*C. ensiformis*	*G. max*	*A. thaliana*
Aldrovanda vesiculosa		79.1	89.5	87.6	76.6	83.9	83.4	74.3	76.2	76.0
Cephalotus follicularis	88.3		80.3	81.0	77.8	80.5	80.1	77.3	80.3	80.4
Dionaea muscipula	94.7	88.8		88.2	76.6	84.1	83.3	75.0	76.4	76.4
Drosera spatulata	93.7	88.3	95.2		77.7	85.7	85.1	74.5	76.1	76.1
Genlisea aurea	87.2	87.2	87.5	87.6		76.2	76.0	72.2	73.7	74.0
Nepenthes alata	91.8	88.8	92.6	92.7	87.5		97.1	75.8	77.2	76.5
Nepenthes hemsleyana	91.6	88.7	92.4	92.5	87.2	98.4		75.0	76.4	75.7
Canavalia ensiformis*	86.0	87.9	86.3	86.5	85.2	86.4	86.0		86.3	74.0
Glycine max**	86.9	89.6	87.4	86.8	85.0	87.5	87.1	93.0		75.3
Arabidopsis thaliana	87.0	89.4	87.2	86.8	84.4	87.1	86.5	85.4	86.0	
**Similarity**										
**Identity**										
* Major isoform										
** Embryo-specific										

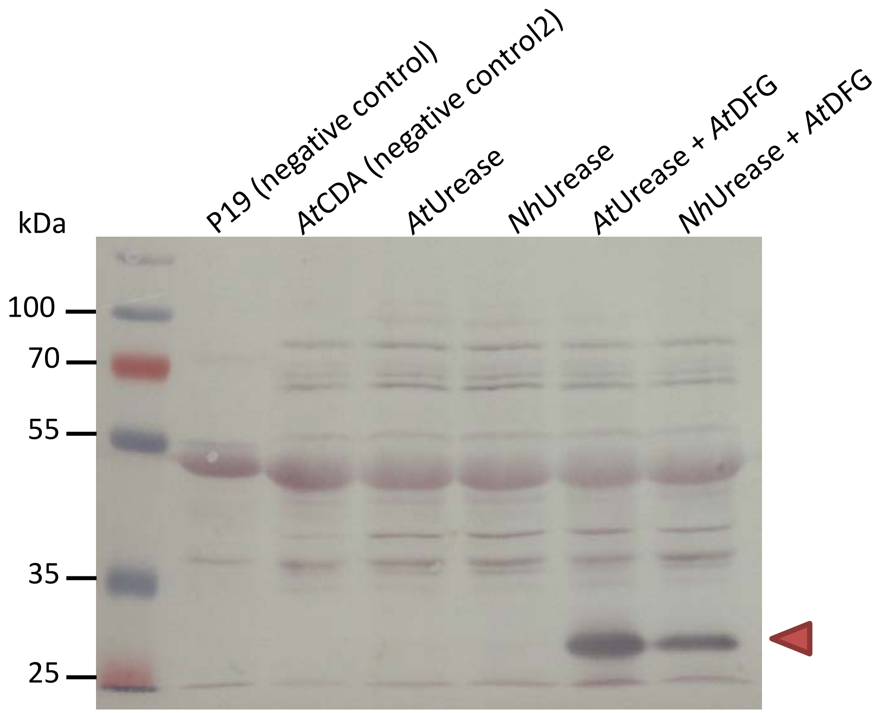

Figure S2. Western blot of *Arabidopsis thaliana At*UreD heterologously co-expressed with urease in *Nicotiana benthamiana*. Full size membrane of the Western blot. 10 µl of each sample was loaded to a 10% SDS gel. The samples are the same as used in Figure 3; additionally with one more negative control of a heterologously expressed CYTIDINE DEAMINASE (*At*CDA) (Ref: Chen et al., 2016, Plant Physiol. 171, 799-809; DOI: 10.1104/pp.15.02031). All lanes were analysed by immunoblot employing specific *At*UreD antiserum. The triangle indicates the targeted protein *At*UreD.

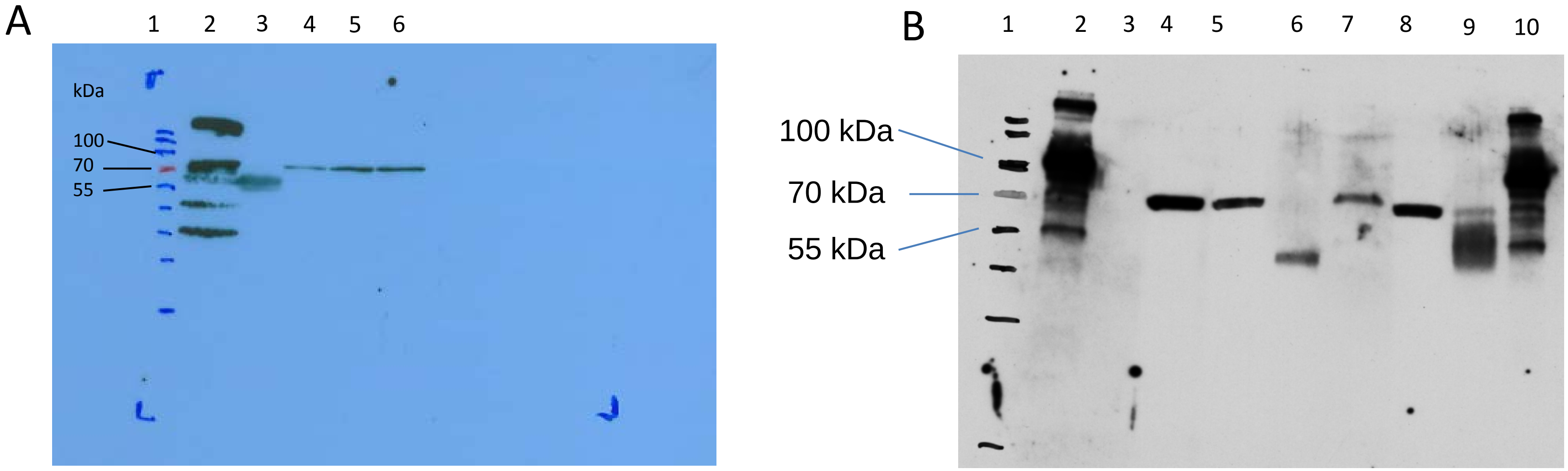

Figure S3. Western blot analyses of urease proteins. Occurrence of urease was done in crude protein extracts from *Nepenthes alata* and *N. hemsleyana* by immunoblot using polyclonal anti-jackbean urease antibodies. (A) Lanes: 1. Page ruler prestained protein ladder (Thermo Fisher); 2. 5 µg total protein extract from soybean seeds; 3. 1 µg of 6 months old degraded total protein extract from *N. alata* pitcher; 4. 1 µg from *N. alata* leaf; 5. 1 µg total protein extract from *N. alata* pitcher; 6. 1 µg total protein extract from another *N. alata* pitcher; Lanes 4, 5, 6 are shown in Figure 2A. (B) Lanes: 1. Page ruler prestained protein ladder; 2. 200 ng urease from *Canavalia ensiformis* (jackbean) as positive control (Sigma-Aldrich); 3. Empty lane; 4. 5 µg total protein extract from *N. hemsleyana* leaf; 5. 5 µg total protein extract from *N. hemsleyana* pitcher; 6. 5 µg, 6 months old degraded total protein extract from *N. hemsleyana* pitcher; 7. 5 µg total protein extract from *N. alata* leaf; 8. 5 µg total protein extract from *N. alata* pitcher; 9. 5 µg, 6 months old degraded total protein extract from *N. alata* pitcher; 10. 200 ng urease from *C. ensiformis*. Lanes 4, 5 are shown in Figure 2A.

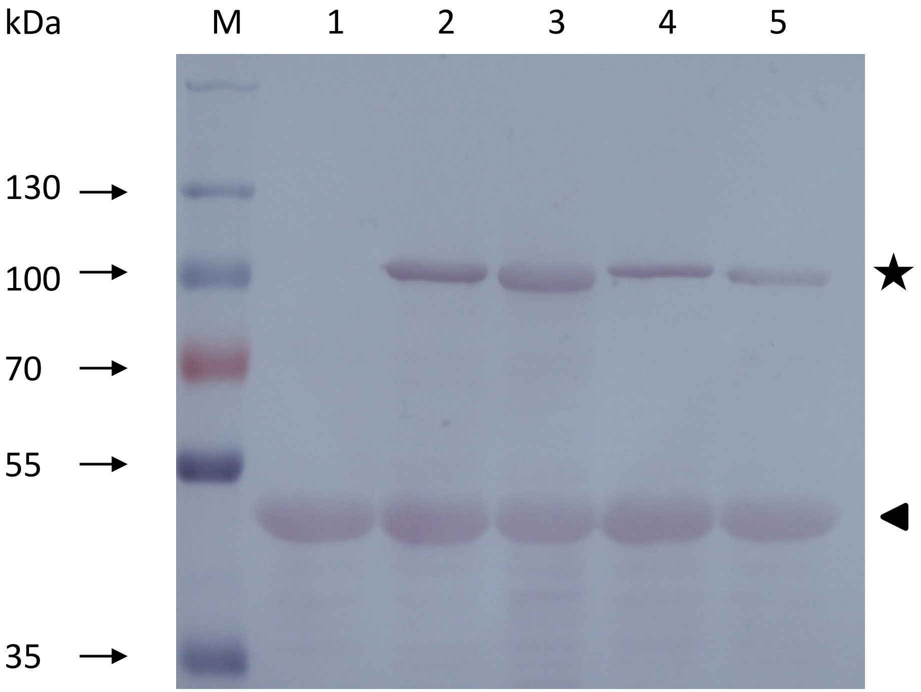

Figure S4. Western blot of *Nepenthes hemsleyana* urease heterologously expressed in *Nicotiana benthamiana*. The full size membrane of the Western blot in Figure 3. Lane 1 to 5 represent extracts of transiently expressed P19: Lane 1 negative control; 2: *A. thaliana* urease; 3: *N. hemsleyana* urease; 4: *A. thaliana* urease with accessory proteins UreD, UreF and UreG; 5: *N. hemsleyana* urease with accessory proteins UreD, UreF and UreG. Western blot was developed with anti *A. thaliana* urease antiserum. The star indicates the targeted protein urease and the triangle indicates Rubisco of *N. benthamiana.*
